# Left-to-Right Shunt with Congenital Heart Disease: Single Center Experience

**DOI:** 10.1155/2013/301617

**Published:** 2013-06-23

**Authors:** Ayhan Cevik, Rana Olgunturk, Serdar Kula, Berna Saylan, Ayhan Pektas, Deniz Oguz, Sedef Tunaoglu

**Affiliations:** ^1^Gazi University Medical Faculty Hospital, Department of Pediatric Cardiology, 06560 Ankara, Turkey; ^2^Dıskapı Children's Hospital Department of Pediatric Cardiology, 06110 Ankara, Turkey

## Abstract

*Objective.* The objective of this study was to determine the frequency of pulmonary arterial hypertension (PAH) in congenital heart disease (CHD) with an isolated, large left-to-right shunt and to indicate the factors in the development of PAH. *Methods.* The pressure measurements in the cardiac chambers and the calculations based on the Fick's principle were compared among 3 separate groups of patients, respectively, with PAH, with hyperkinetic pulmonary hypertension (HPH), and with neither PAH nor HPH. *Results.* PAH was diagnosed in 30 (12.3%) patients, HPH in 35 (14.4%), while 177 (73.1%) were free of either. The highest risk for the development of PAH was found in the presence of perimembranous ventricular septal defect. A statistically significant difference was seen among these groups as to their left atrial pressure (*p* = 0.005) and the mean pulmonary arterial pressure (PAP_mean_; *p* < 0.001). While a correlation was present between RpI on one hand and age on the other (*p* = 0.014), a multiple linear regression could not evidence any correlation among age (*p* = 0.321), gender (*p* = 0.929). *Conclusion.* Our findings do not allow establishing a correlation between the duration of the high pulmonary flow and pulmonary vascular resistance increase or PAH development in isolated left-to-right shunts with congenital heart diseases.

## 1. Introduction

In all congenital heart diseases (CHD) with unrestricted left-to-right shunt, high pulmonary arterial flow can present together with pulmonary arterial hypertension (PAH); the grade of pulmonary vascular lesions is, however, important in the development of pulmonary vascular lesions [1]. The most important, in frequency, of the conditions leading to PAH in CHD are those which include left-to-right shunts [2]. While estimating the risk of PAH is of primordial importance for pediatric patients with a left-to-right shunt, there is no consensus or established guideline to date [3]. How is the risk of developing PAH in cases of CHD with left-to-right shunt characterized, and which are the determining factors? Several sets of criteria and practices have been developed regarding surgical indication and prognosis; the accepted gold standard in diagnosis is right heart catheterization [4]. This invasive technique, however, does not lend itself to easy, repeated use. Additionally, the calculation of indices for cardiac catheterization based on the Fick's principle does not necessarily correspond to the reality. Stimuli such as increased pulmonary arterial flow and pressure lead to an imbalance in vascular tonus and to vascular remodeling [5]. The degree of pulmonary hyperemia, the type of cardiac lesion, and the defect dimensions seem to be, according to published reports, the main factors in the development of irreversible pulmonary vascular damage in septal defect patients with left-to-right shunt [6]. Studies in CHD patients on their risk of developing PAH have shown the presence of a genetic mutation of type II bone morphogenic protein (BMPR2) in 6% only of this population. Mutations belonging to the TGF-*β* super family have infrequently been reported in forms of PAH [7]. Considering that approximately one third of CHD patients who do not undergo surgical correction are predicted to develop PAH, a correct evaluation of this risk would play a major role in determining morbidity and mortality.

The objective of this study was to determine the frequency of PAH in our CHD patients without genetic pathology who had undergone cardiac catheterization for an isolated, large left-to-right shunt and to characterize the variables correlating with RpI. Identifying the parameters which correlate with the pulmonary vascular resistance index could provide very helpful indications for the management and the prognosis of these patients.

## 2. Methods

### 2.1. Study Design and Population

A total of 242 patients aged 4–198 months with a diagnosis of CHD and isolated, large left-to-right shunt who had been seen between October 2006 and November 2011 at the Pediatric Cardiology Department of the Gazi University Medical School were included in this retrospective evaluation. The cases were evaluated in three separate groups. Group I consisted of patients with a diagnosis of PAH. Criteria for the diagnosis of PAH were a mean pulmonary artery pressure (PAP_mean_) > 25 mmHg, pulmonary capillary wedge pressure (PCWP) < 15 mmHg, pulmonary vascular resistance index (RpI) > 3 WU/m² (250 dyn*·*s*·*cm^−5^) [7]. PH in group II was defined as hyperkinetic pulmonary hypertension (HPH) for a PAP_mean_ of ≥25 mmHg as measured at rest during right heart catheterization and RpI < 3 WU/m². Patients with a PAP_mean_ < 25 mmHg measured as described above were assigned to group III [8, 9].

Patients who had been evaluated by invasive hemodynamic diagnostics by cardiac catheterization in view of an intervention necessitated by heart failure and cardiomegaly were included in the surgical treatment plan, as well as patients who fulfilled the criteria decribed below. The inclusion criterion for the presence of a large left-to-right shunt at evaluation by transthoracic echocardiography (TTE) was a diameter of >2 cm for an atrial septal defect (ASD) and >1 cm for a ventricular septal defect (VSD) [10]. The inclusion criterion for the patent ductus arteriosus (PDA) was the presence of a left-to-right shunt at ductal level by TTE and the presence of the characteristic murmur for the PDA on physical examination. Criteria for exclusion from the study were the following: patients given general anesthesia for cardiac catheterization, those necessitating oxygen therapy and respiratory support, patients with additional systemic disease or genetic pathology such as Down's syndrome, presence of a general condition which could negatively impact hemodynamic determinations, patients with low cardiac output, those receiving treatment for congestive heart failure, patients with aneurysm, and those with pouch formation.

### 2.2. Cardiac Catheterization

Premedication for cardiac catheterization was performed with intravenous midazolam, 0.1 mg/kg; patients who received additional sedative or anesthetic agents were excluded from the study in order to avoid measurement errors on which to base calculations after the Fick's principle. Pressure recordings and blood samples were obtained from the heart chambers and the large vessels in all patients following catheterization of femoral vein and artery. Pulmonary blood flow (Qp) and systemic blood flow (Qs) was calculated on the basis of Fick's principle. Hemodynamic measurements consisted of left atrial pressure, left atrial pressure or PCWP, systolic, diastolic and mean pulmonary artery pressures, pulmonary and systemic blood flow, flow ratio, pulmonary and systemic resistance, and resistance ratio. These values were divided by the body surface area for use as standardized indexes. Estimated values according to age, sex, and heart rate were used for oxygen consumption.

### 2.3. Statistical Analysis

Data analysis was performed using SPSS for Windows, version 15.0. The normality of continuous variable distribution was tested by the Shapiro-Wilk test. Homogeneity of variance was evaluated by the Levene test. Data were expressed either as mean ± 1 SD or median (minimum-maximum), as appropriate. Differences in the group means were evaluated using one-way analysis of variance (ANOVA), while differences among median values were evaluated by the Kruskal-Wallis test. Tukey's post hoc procedure or Conover's multiple comparison test was used in case of a statistically significant result of ANOVA or Kruskal-Wallis tests to determine the different groups. Categorical variables were evaluated using Pearson's chi-squared test. After correcting for age and sex, the continuation of the effect on RpI in both the PAH and PH groups was evaluated by multivariable linear regression analysis. A *P*-value inferior to 0.05 was considered statistically significant.

## 3. Results

A total number of 242 subjects were included in the study. Their average age was 61.6 ± 51.2 months. The patient's number, central values, and age range for the three groups were as follows: group I, 30 patients aged 32.5 ± 46.4 months (6–156); group II, 35 patients aged 25.9 ± 33.2 months (6–132); group III, 177 patients aged 73.7 ± 49.8 months (6–216).

There was no significant difference between the first two, while age in group III was higher than in both of these (Table 1). Even though there seemed to be more females in group I and more males in group II (66.7 and 57.1%, respectively), there was no statistically established difference. There were 66 boys for 111 girls (37.3 and 62.7%, resp.) in group III.

The most frequent anatomic condition in both groups I and II was perimembranous VSD, while in Group III it was ASD (Table 2). PAH was found in 30 (12.3%) of 242 patients. Of these, 19 (63.3%) are being followed with a VSD diagnosis.

There was no difference among groups as to Qp (7.5, 8.2 and 6.4 L/min/m², resp.). Qs, on the other hand, differed among the three groups. Qp, Qs, and the Qp/Qs ratio were not found to be a determining factor in the development of PAH. There was no statistically detectable difference between Groups I and II for RsI, RpI/RsI, or left atrial pressure even though there was a perceived difference (Table 3).

Stepwise multivariable linear regression analysis of all those variables that had a statistically significant correlation with RpI (Table 4) showed that the independent variable most significantly influencing change in RpI was age (*P* = 0.014 with a 95% confidence interval of 0.011–0.074).

After correcting for age and sex, the significant effect on RpI disappeared (*P* = 0.321, *P* = 0.929), while the significant elevation of RpI in both the PAH and PH groups remained. The results of multivariable linear regression analysis are shown in Table 5.

## 4. Discussion

Correct management of heart defects with left-to-right shunt is important in the prevention of progressive pulmonary vascular disease. It is estimated that pulmonary vascular disease develops in 15% (10–18%) of all CHD. The uncorrected CHD defects in these cause a left-to-right shunt; the resulting continual increase in pulmonary flow increases tensile stress, which leads to an elevation of Rp and finally to pulmonary vascular damage [11]. In the initial stage of CHD with left-to-right shunt, PAP increases due to the higher Qp that follows the fall in Rp during the postnatal period. This is the stage of hyperkinetic PH, caused only by increased flow in the presence of low RpI. The structural changes resulting from the long-term tensile stress due to increased flow in the pulmonary vascular bed are luminal narrowing and the consequent resistance increase. Irreversible change takes place in the advanced stages of this process [12]. In our review, the average age of the patients who developed PAH and PH was, respectively, 32.5 and 25.9 months; there was no correlation between age and the onset of the respective conditions. A predominance of girls, with 66.7%, was observed among PAH patients in our study, consistent in this with the published literature [5].

The estimated prevalence, in developed countries, of PAH in adults with CHD including a left-to-right shunt is 1.6–12.5 cases per million [8]. According to data of the Dutch CONCOR National Registry, PAH develops in 6.1% of cases with septal defect [9]. No information is available on the incidence or prevalence of PAH accompanying CHD in children. The frequency of PAH in all our patients with septal defect was 30 in 242 (12.3%) This higher than expected proportion may have been due to the inclusion of patients with large defects and isolated left-to-right shunts.

The size and location of the septal defect are an important determinant of PAH in the presence of CHD both in children and adults. Simple defects are considered in two groups according to location, pretricuspid and posttricuspid shunts [5]. Pretricuspid lesions (ASD) are associated with a lateronset and a lower-frequency PAH in response to the increase in pulmonary circulation compared to the posttricuspid shunts (VSD, PDA). Posttricuspid shunts, VSD and PDA, lead to both pressure and volume overload. Defect size is of importance equal to that of location. Patients with large defects are more prone to development of PAH than those with smaller ones. PAH develops in only 3% of small and middle-sized VSD, while it reaches up to 50% in frequency with large defects [6]. Small defects are defined as <1 cm for VSD and <2 cm for ASD [10]. Perimembranous VSD was the most frequently found condition, in 19 of 30 patients (63.3%) who developed PAH in our study. PAH was not found in patients with either secundum ASD or muscular VSD. This absence, in the presence of large defects, suggested that the risk of developing PAH with these two conditions is low. Thus, according to our study the condition with the highest risk of developing PAH is perimembranous VSD. Patients with the latter condition should be closely followed up and treated early. PDA was second in frequency with 6 of 30 patients with PAH (20%). It was interesting to note the less frequent occurrence of PAH in patients with multiple defects as compared to those with a single defect. Patients with multiple defects developed PAH in 5 cases (16.6%; VSD+ASD 1, VSD+PDA 2, VSD+ASD+PDA 2 cases). According to our findings, even though the presence of multiple defects, somewhat increases the risk, it does not constitute as much of an additional danger as expected.

Pulmonary resistance index (RpI) cannot be measured directly; it is calculated by the ratio of pulmonary pressure variation to pulmonary arterial flow. While right heart catheterization measurements are helpful, in fact the best available tool to date, they are not always reliable. Even patients who are in the interval recognized as predicting a good outcome of surgery may show a persistent postoperative PAH. More manageable and less invasive tools are needed, especially for patients who are a borderline for operability according to their hemodynamic profile. Lévy et al. report recent developments which could justify hopes of new tools for determining operability [13]. The adequacy of a vasoreactivity test alone to determine the operability of patients with CHD and high RpI is controversial as to its predictivity of surgical success. Technical difficulties which may lead to a wrong calculation, as well as concomitant medical conditions, should be borne in mind when performing a vasodilator test. The question of which preoperative pulmonary hemodynamic parameters best correlate with surgical outcome remains open.

Published literature indicates that hemodynamic values obtained by cardiac catheterization are the principal prognostic factor. An analysis based on records of the US National Institute of Health (NIH) shows a particularly high prognostic importance of three hemodynamic variables for survival [14]. These are increased PAP_mean_, increased mean right atrial pressure, and reduced cardiac index. No significant difference was seen between Groups I and II with regard to PAP_mean_, systolic and diastolic pulmonary arterial pressure (PAP_syst_, PAP_diast_) and left atrial pressure. As a result, these parameters were not considered risk factors for the development of PAH. The significant single-pair correlations shown in Table 4 for RpI with age and gender were not confirmed by the later multivariable regression analysis; it was concluded that in our study RpI was not correlated to these variables.

The influence of individual conditions like the type of lesion and genetic predisposition on the result remained incompletely understood. Studies have been performed on the prognostic value of other investigations than catheterization, including lung biopsy, a more rarely used invasive procedure, and the high risk of irreversible PAH after CHD correction indicated by circulating endothelial cells as reported by Smadja et al. [15, 16].

Along with intimal thickening of the pulmonary artery and increased bcl-2 expression in endothelial cells in lung biopsy, patients with irreversible PAH seemed to have a higher number of circulating endothelial cells than those with a reversible disease. Findings other than CEC, like endothelial activation, regeneration, and injury biomarkers, do not differentiate between reversible and irreversible PAH.

Finally, it is not possible as of today to predict exactly when and under the influence of which risk factors PAH is likely to develop; numerous studies including many more cases are needed to understand this complex disease.

As for the limitations of the study, the lack of genetic marker data of the patients is an important one, even though, with the knowledge of studies performed to date, genetic markers were found in small numbers in nonhereditary PAH patients. Such determinations are not yet at the point of being part of the routine evaluation of patients with left-to-right shunts. Another limitation is the fact that, even though vasoreactivity had been tested in our PAH patients, it has not been presented as various methods, and various agents had been employed for the test.

In conclusion, patients followed up with a diagnosis of VSD are the most at risk for PAH among those with isolated large left-to-right shunt. PAH developed in 12.3% of cases of isolated left-to-right shunt. A correlation could not be established between pulmonary vascular resistance increase, provoked by the patient's age or the exposure duration of the pulmonary vascular bed and the development of PAH. Considering the probable effect of factors other than age, particular attention should be paid to following up these cases for the development of irreversible pulmonary vascular remodeling, a situation to be kept in mind also in determining the follow-up periods.

## Figures and Tables

**Table 1:** Demographic characteristics of the subjects by groups.

Variables	Group I (Control)		Group II		Group III		*P*-value
	*n*	%	*n*	%	*n*	%	
	30	12.3	35	14.4	177	73.1	
Age (months)	32.5 ± 46.4^a^		25.9 ± 33.2^b^		73.7 ± 49.8^a,b^		**<0.001**
Gender							0.068
Male	10 (33.3%)		20 (57.1%)		66 (37.3%)		
Female	20 (66.7%)		15 (42.9%)		111 (62.7%)		

^a^Statistically significant differences between groups I and III (*P* < 0.01), ^b^statistically significant differences between groups II and III (*P* < 0.05).

**Table 2:** Distribution of Disease Diagnosis by Groups.

Diagnosis	Group I		Group II		Group III		Total	
	*n* = 30	%	*n* = 35	%	*n* = 177	%	*n* = 242	%
ASD	0	0.0	1	2.9	65	36.7	66	27.3
VSD (PM)	19	63.3	15	42.9	46	26.0	80	33.1
VSD (muscular)	0	0.0	0	0.0	2	1.1	2	0.8
PDA	6	20.0	10	28.6	48	27.1	64	26.4
VSD (PM) and ASD	1	3.3	5	14.3	2	1.1	8	3.3
VSD (PM) and PDA	2	6.7	3	8.6	9	5.1	14	5.8
VSD (PM) and ASD and PDA	2	6.7	1	2.9	0	0.0	3	1.2
ASD and PDA	0	0.0	0	0.0	5	2.8	5	2.1

Total	30	100.0	35	100.0	177	100.0	242	100

ASD: atrial septal defect, PDA: patent ductus arteriosus, PM: perimembranous, VSD: ventricular septal defect.

**Table 3:** Hemodynamic findings at cardiac catheterization by groups.

Variable	Group I	Group II	Group III	*P* value
	*N* = 30	*N* = 35	*N* = 177	
RAP (mmHg)	5 (2–12)	5 (3–17)	5 (1–13)	0.329
LAP (mmHg)	10 (7–15)^a^	9 (5–17)^b^	7 (1–17)^a,b^	**0.005**
PAP_mean_ (mmHg)	43 (27–96)^a^	33 (25–57)^b^	16 (9–26)^a,b^	**<0.001**
PAP_systolic_ (mmHg)	66 (40–126)^a^	52 (30–78)^b^	25 (14–63)^a,b^	**<0.001**
PAP_diastolic_ (mmHg)	23 (4–76)^a^	17 (7–37)^b^	9 (1–19)^a,b^	**<0.001**
Aort_mean_ pressure (mmHg)	70.0 ± 13.4	70.7 ± 13.2	77.9 ± 14.3	0.058
Aort_systolic_ (mmHg)	92.2 ± 15.5	91.5 ± 16.9	98.2 ± 13.5	0.078
Aort_diastolic_ (mmHg)	52.9 ± 11.4	51.5 ± 10.4	57.4 ± 13.3	0.082
Qp (L/min/m²)	7.5 (1.9–22.1)	8.2 (3.0–29.0)	6.4 (2.4–19.9)	0.124
Qs (L/min/m²)	3.2 (0.9–5.1)^a^	3.5 (0.8–7.4)^b^	3.9 (2.0–7.4)^a,b^	**0.007**
Qp/Qs	2.4 (1.1–9.2)^a^	2.6 (1.3–5.7)^b^	1.6 (0.6–6.5)^a,b^	**<0.001**
RpI (WU/m²)	11.0 (3.2–20.0)^a,c^	2.6 (1.0–6.6)^b,c^	1.1 (0.4–3.0)^a,b^	**<0.001**
Rs (WU/m²)	19.7 (12.0–27.0)	15.5 (12.0–28.2)	20.0 (11.0–27.7)	0.731
RpI/RsI	2.2 (0.2–8.3)^a^	2.3 (0.05–9.0)^b^	1.2 (0.01–3.8)^a,b^	**<0.001**

Statistically significant differences between ^a^groups I and III (*P* < 0.01); ^b^II and III (*P* < 0.05); ^c^I and II (*P* < 0.01).

LAP: left atrium pressure, PAP: pulmonary arterial pressure, Qp: pulmonary arterial flow, RAP: right atrium pressure, RpI: pulmonary resistance index, RsI: systemic resistance index, Qs: systemic blood flow.

**Table 4:** Correlation of RpI with other variables.

Variable	RpI	
	*r*	*P* value
Age	0.422	**0.014**
Gender	0.132	0.548
RAP	0.017	0.926
LAP	0.600	0.208
PCWP	0.335	0.241
Aort (mean) pressure	−0.136	0.569
RsI	−0.011	0.956

LAP: left atrium pressure, PCWP: pulmonary capillary wedge pressure, RAP: right atrium pressure, RsI: systemic resistance index.

**Table 5:** Correlations with RpI in a multivariable linear regression analysis.

Variable	Regression coefficient	*P* value	95% confidence interval	
			Lower limit	Upper limit
Age	−0.001	0.321	−0.002	0.001
Gender	0.005	0.929	−0.099	0.109
